# Factors Associated With Abrupt Discontinuation of Long-Term High-Dose Opioid Treatment

**DOI:** 10.1001/jamanetworkopen.2023.41416

**Published:** 2023-11-03

**Authors:** Carolina Vivas-Valencia, Huiru Dong, Erin J. Stringfellow, W. Alton Russell, Jake R. Morgan, Mina Tadrous, Mohammad S. Jalali

**Affiliations:** 1 Department of Biomedical Engineering and Chemical Engineering, The University of Texas, San Antonio; 2 Massachusetts General Hospital, Harvard Medical School, Boston; 3 Department of Epidemiology, Biostatistics and Occupational Health, McGill University, Montreal, Quebec, Canada; 4 Department of Health Law, Policy, and Management, Boston University School of Public Health, Boston; 5 Leslie Dan Faculty of Pharmacy, University of Toronto, Toronto, Ontario, Canada

## Abstract

This cohort study investigates factors associated with abrupt discontinuation of long-term high-dose opioid treatment at the national level and across US states.

## Introduction

Managing long-term high-dose (LTHD) opioid regimens is a concern.^1^ The repercussions of abrupt discontinuation are profound, encompassing adverse outcomes such as withdrawal, illicit substance use, and mental health complications.^2,3^ Research shows that a notable majority of patients, exceeding 70% nationwide, encounter abrupt discontinuation of LTHD treatments,^4^ which signals potential gaps in management strategies. This study aims to identify factors associated with abrupt discontinuation, extending previous research^4,5,6^ by incorporating more recent data and identifying their associations at the national level and across states.

## Methods

This cohort study followed the STROBE reporting guideline and was exempt from review and granted waiver of informed consent by the Mass General Brigham institutional review board because data were not obtained through participant interaction. We analyzed a sample from the IQVIA Longitudinal Prescription database to identify opioid treatment episodes that started in January 2014 or later and had been discontinued by September 2020. We examined all data for the following year (through September 2021) to ensure participants remained continuously enrolled. LTHD episodes were defined as lasting at least 90 days with at least 90 daily morphine milligram equivalents (DMME) for at least 14 consecutive days.^4^ Episodes were abruptly discontinued if the final 30 days’ DMME was at least 60 or represented a greater than 35% reduction compared with the previous 30 days (eMethods in Supplement 1). Associations between abrupt discontinuation and race and ethnicity, age, sex, payment source, initial opioid dosage (included to control for clinical decisions), and year of discontinuation were analyzed using multivariable logistic regressions at national and state levels. Race and ethnicity data were collected to identify disparities among different races and ethnicities.

Study design details are available in the eMethods in Supplement 1. Statistical analysis was performed using R software version 4.2.0 (R Project for Statistical Computing) from September 2022 to May 2023.

## Results

Among 354 940 discontinued LTHD episodes, 192 037 (54.1%) were from female patients; 151 352 (42.6%) were aged at least 65 years, 135 784 (38.3%) were aged 50 to 64 years; 46 332 (13.1%) were Black, 24 535 (6.9%) were Hispanic, and 284 073 (80.0%) were White. During the study period, 247 308 (69.7%) of discontinued episodes were abruptly discontinued. Nationally, abrupt discontinuation was positively associated with being male, Black, Hispanic, or aged at least 50 years; having Medicare, private insurance, or being cash-paying; and starting with DMME greater than or equal to 50. The rate of abrupt discontinuation also decreased between 2014 and 2019 (Table).

**Table.** Patient Characteristics and Associations With Abrupt Discontinuation of Long-Term High-Dose Opioid Treatment Episodes, Stratified by Episode Discontinuation Type at the National Level

Variables	No. (%)			Odds ratio (95% CI)	
	Discontinued LTHD episodes (N = 354 940)	Abruptly discontinued episodes (n = 247 308)	Gradually discontinued (n = 107 632)	Unadjusted	Adjusted
Sex					
Female	192 037 (54.1)	132 050 (53.4)	59 987 (55.7)	1 [Reference]	1 [Reference]
Male	162 903 (45.9)	115 258 (46.6)	47 645 (44.3)	1.09 (1.08-1.11)	1.09 (1.08-1.11)
Age, y					
18-34	9964 (2.8)	6677 (2.7)	3287 (3.1)	1 [Reference]	1 [Reference]
35-49	57 840 (16.3)	39 475 (16.0)	18 365 (17.1)	1.05 (1.01-1.10)	1.04 (0.99-1.09)
50-64	135 784 (38.3)	94 163 (38.1)	41 621 (38.7)	1.11 (1.06-1.16)	1.08 (1.03-1.13)
≥65	151 352 (42.6)	106 993 (43.3)	44 359 (41.2)	1.18 (1.13-1.23)	1.16 (1.11-1.22)
Race and ethnicity					
Black	46 332 (13.1)	32 827 (13.3)	13 505 (12.5)	1.07 (1.04-1.09)	1.08 (1.06-1.11)
Hispanic	24 535 (6.9)	17 243 (7.0)	7292 (6.8)	1.04 (1.01-1.07)	1.06 (1.03-1.09)
White	284 073 (80.0)	197 238 (79.8)	86 835 (80.7)	1 [Reference]	1 [Reference]
Payment					
Medicaid	10 304 (2.9)	6883 (2.8)	3421 (3.2)	1 [Reference]	1 [Reference]
Medicare	135 758 (38.2)	95 854 (38.8)	39 904 (37.1)	1.19 (1.14-1.24)	1.11 (1.06-1.16)
Private insurance	188 967 (53.2)	129 569 (52.4)	59 398 (55.2)	1.08 (1.03-1.13)	1.06 (1.02-1.11)
Cash	19 911 (5.6)	15 002 (6.1)	4909 (4.6)	1.52 (1.44-1.60)	1.40 (1.33-1.48)
Initial DMME level					
<50	97 396 (27.4)	57 877 (23.4)	39 519 (36.7)	1 [Reference]	1 [Reference]
50-90	141 794 (39.9)	109 857 (44.4)	31 937 (29.7)	1.34 (1.31-1.37)	1.33 (1.31-1.36)
90-120	59 788 (16.8)	39 639 (16.0)	20 149 (18.7)	1.70 (1.66-1.73)	1.68 (1.64-1.72)
≥120	55 962 (15.8)	39 935 (16.1)	16 027 (14.9)	2.34 (2.30-2.39)	2.30 (2.26-2.34)
Year of discontinuation					
Per year increase	NA	NA	NA	0.94 (0.93-0.94)	0.95 (0.94-0.95)

Abbreviations: DMME, daily morphine milligram equivalents; LTHD, long-term high-dose; NA, not applicable.

In 9 states, Black patients had a higher likelihood of abrupt discontinuation than White patients, and a lower risk in 1 state (Figure); Hispanic patients had a higher risk in 2 states. Abrupt discontinuation was more likely among male patients in 12 states (Figure). Compared with Medicaid enrollees, cash payers faced a higher risk in 6 states. In only 1 state were patients with private insurance at higher risk, whereas Medicare patients were at higher risk in 2 states. Starting with DMME greater than or equal to 120 was associated with increased risk of abrupt discontinuation in 48 states and the District of Columbia. Twenty-nine states had a decrease over time in abrupt discontinuations.

**Figure.**
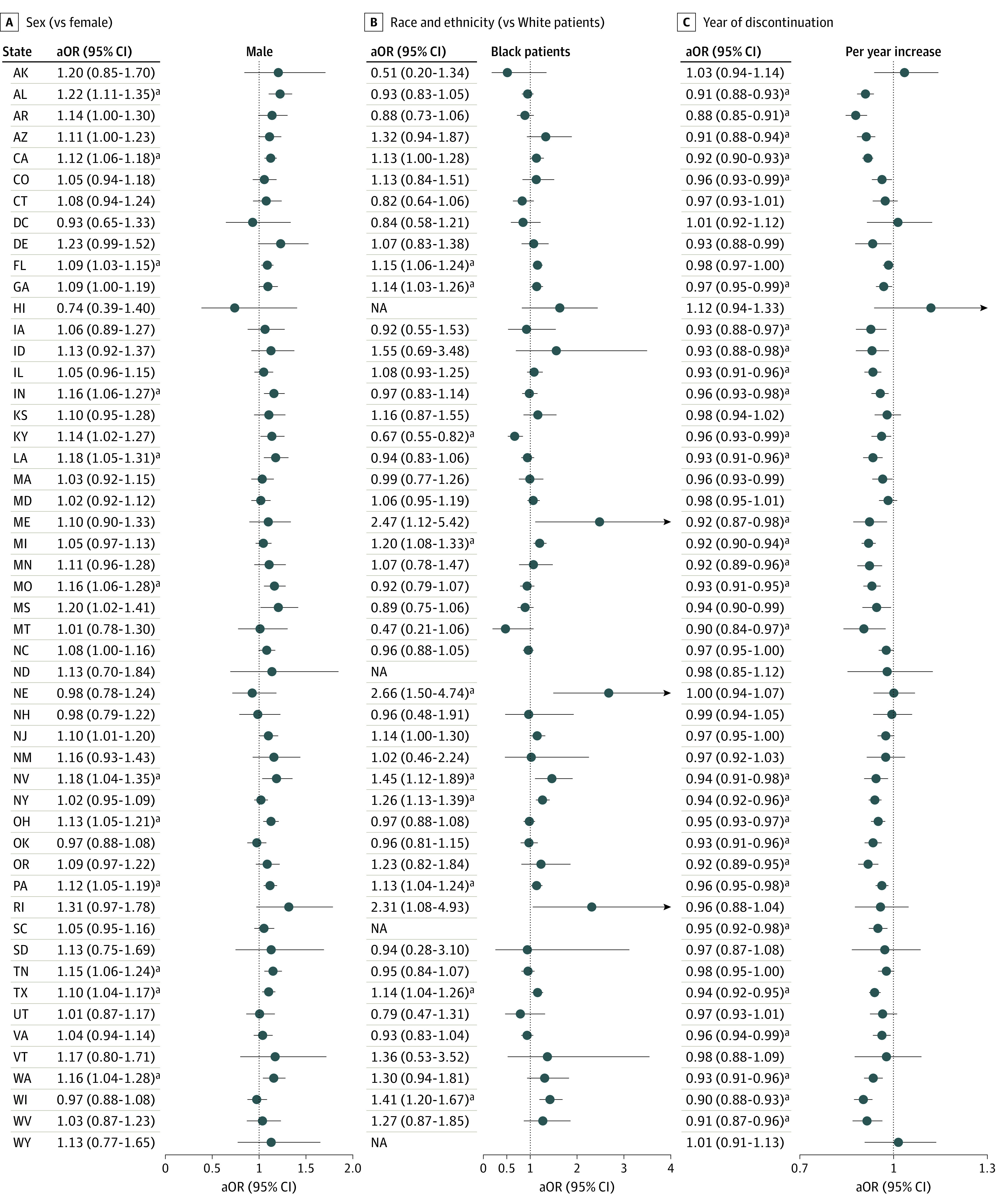
Factors Associated With Abrupt Discontinuation of Long-Term High-Dose Opioid Treatment Episodes at the State Level Variables were not included in the state-level models if any categories had low prevalence due to small sample sizes. Other statistically significant results not presented in the figure include Arizona and New York (Hispanic patients); California and South Carolina (aged 50-64 years); California, Colorado, Idaho, Indiana, Nebraska, Ohio, and South Carolina (aged 65 years or older); Alaska, California, Delaware, Florida, Michigan, and Tennessee (cash insurance); Alaska (private insurance); and Alaska and Arizona (Medicare payment). For DMMEs, 50 to 90 was significant in 32 states, 90 to 120 was significant in 35 states, and 120 or greater was significant 49 states. aOR indicates adjusted odds ratio; NA, not applicable. ^a^Significant result after Benjamini–Hochberg correction for multiple comparisons.

## Discussion

Our national-level cohort study identified a significant risk of abrupt discontinuation, with the associated sociodemographic factors consistent with prior research.^4^ We also found that abrupt discontinuation occurred less frequently over time, suggesting that clinicians have responded to concerns about the risks associated with this practice. Additionally, our study found large heterogeneities across states, which were distinct from the national-level results. At the state level, sociodemographic factors most often associated with abrupt discontinuation were being male and a Black patient.

A limitation of our study is the lack of data on rationale for dosage adjustments, treatment discontinuation, and information on patient behavior, especially whether having received guidance from the physician on tapering within the last high-dosage prescription. However, we would not expect Black or male patients to consistently have conditions or practices that would make abrupt discontinuation more appropriate for them than for White or female patients. Thus, it is likely our findings point to real disparities. Further analysis should investigate the reasons for the disparities and why they exist in some states but not others.

## Notes

Supplement 1. **eMethods. **Supplemental Methods
eReferences

Supplement 2.
Data Sharing Statement

## References

[1] Baldini A, Von Korff M, Lin EHB. A review of potential adverse effects of long-term opioid therapy: a practitioner’s guide. Prim Care Companion CNS Disord. 2012;14(3):PCC.11m01326. doi:10.4088/PCC.11m01326 23106029 PMC3466038

[2] Oliva EM, Bowe T, Manhapra A, . Associations between stopping prescriptions for opioids, length of opioid treatment, and overdose or suicide deaths in US veterans: observational evaluation. BMJ. 2020;368:m283. doi:10.1136/bmj.m283 32131996 PMC7249243

[3] Binswanger IA, Glanz JM, Faul M, . The association between opioid discontinuation and heroin use: a nested case-control study. Drug Alcohol Depend. 2020;217:108248. doi:10.1016/j.drugalcdep.2020.108248 32927194 PMC10959283

[4] Stein BD, Sherry TB, O’Neill B, Taylor EA, Sorbero M. Rapid discontinuation of chronic, high-dose opioid treatment for pain: prevalence and associated factors. J Gen Intern Med. 2022;37(7):1603-1609. doi:10.1007/s11606-021-07119-3 34608565 PMC9130349

[5] Bao Y, Wen K, Johnson P, Witkin LR, Reid MC. Abrupt discontinuation of long-term opioid therapies among privately insured or Medicare Advantage adults, 2011-2017. Pain Med. 2021;22(7):1702-1704. doi:10.1093/pm/pnaa350 33155020 PMC8311577

[6] Neprash HT, Gaye M, Barnett ML. Abrupt discontinuation of long-term opioid therapy among Medicare beneficiaries, 2012-2017. J Gen Intern Med. 2021;36(6):1576-1583. doi:10.1007/s11606-020-06402-z 33515197 PMC8175547

