# Supplementary material for: Factors Associated With Abrupt Discontinuation of Long-Term High-Dose Opioid Treatment
Source: JAMA Netw Open. 2023 Nov 3;6(11):e2341416. doi: 10.1001/jamanetworkopen.2023.41416 (PMC10625034; doi:10.1001/jamanetworkopen.2023.41416)
Supplement: Supplement 1. — eMethods. Supplemental Methods eReferences [file jamanetwopen-e2341416-s001.pdf]

Supplemental Online Content

Vivas-Valencia C, Dong H, Stringfellow EJ, et al. Factors associated with abrupt discontinuation of long-term high-dose opioid treatment. *JAMA Netw Open.* 2023;6(11):e2341416. doi:10.1001/jamanetworkopen.2023.41416

eMethods. Supplemental Methods
eReferences

This supplemental material has been provided by the authors to give readers additional information about their work.

© 2023 Vivas-Valencia C et al. *JAMA Network Open.*

eMethods. Supplemental Methods

Here we describe additional details about the methodology used**.**

Study Data and Population
In this cohort study, we used a sample from IQVIA longitudinal prescription (LRx) and medical claims (Dx) databases. The population coverage of LRx has increased over time. In 2021, it covered 93% of prescriptions from dispensed retail channels, 77% from traditional and specialty mail orders, and 74% from long-term care. It contains information regarding the marketed product names, the quantity dispensed for each prescription, and the number of days the prescription lasts. The Dx database comprises procedures and diagnosis information collected annually from office-based physicians and specialists. Patient demographic data include age, sex, race/ethnicity, payment type, and three-digit ZIP codes.

To identify the list of opioid analgesics between January 2014 and September 2021, we used the information available in the RED BOOK database (Series, Micromedex Healthcare. "Red Book Online." (2019)). The final list, reported below, was reviewed by a group of experts, including, pharmacists, medical doctors, and researchers in the area of opioid use disorder.

> Acetaminophenw/codeine, acetaminophen-codeine#4, acetaminophen/codeine, acetaminophen/codeine#2, acetaminophen/codeine#3, acetaminophen/codeinepho, acetaminophen/oxycodone, aspirin-caffeine-dihydroc, benzhydrocodone/acetamino, butorphanoltartrate, capitalandcodeine, capital/codeine, celecoxib, codeinesulfate, fentanyl, fentanylcitrate, fentanylcitrateoraltra, fentanyloralet, fentanyl-ropivacaine-0.9%nacl, fentanyl/ns, hydrocodonebitartrate, hydrocodonebitartrateer, hydrocodonebitartrate/ac, hydrocodonebitartrate/ap, hydrocodonebitartrate/ho, hydrocodonecompound, hydrocodonepolistirex/ch, hydrocodone-acetaminophen, hydrocodone/acetaminophen, hydrocodone/apap, hydrocodone/guaifenesin, hydrocodone/ibuprofen, hydromorphonehcl, hydromorphonehcler, hydromorphonehcl-0.9%nacl, hydromorphonehcl/sodium, hydromorphonehydrochlori, levorphanoltartrate, meperidinehcl, meperidinehcltubex, meperidinehcl/promethazi, meperidine/promethazine, methadonehcl, methadonehcldiskets, methadonehclintensol, methadonehydrochloride, methadonehydrochloridei, morphinesulfate, morphinesulfateadd-vant, morphinesulfatecr, morphinesulfatedilute-a, morphinesulfateer, morphinesulfatefliptop, morphinesulfateindextr, morphinesulfateir, morphinesulfatestick-ga, morphinesulfate-0.9%nacl, morphinesulfate/ns, morphinesulfate/sodiumc, oxycodonehclcr, oxycodonehcler, oxycodonehydrochloride, oxycodonehydrochloridee, oxycodone/acetaminophen, oxycodone/apap, oxycodone/aspirin, oxycodone/ibuprofen, pentazocine/acetaminophen, pentazocine/naloxonehcl, phenaphen/codeine#4, somacompound/codeine, tramadolhcl, tramadolhcler, tramadolhydrochloride, tramadolhydrochlorideer, tramadolhydrochloride/ac, tylenol/codeine, tylenol/codeine#3, tylenol/codeine#4, abstral, acetaminophen/codeine#4, acetaminophen-codeinephosphate, actiq, arymoer, avinza, butrans, codeine, codeinephosphate, codeinephosphate/guai, codeinephosphate/guaifenesin, conzip, darvon, darvoncompound31, darvoncompound-65, darvon-n, demerol, demerolapap, demerolhydrochloride, dilaudid, dilaudid-5, dilaudid-hp, dolophinehcl, duragesic, duramorph, embeda, endocet, endocodone, exalgo, fentanylbase, fentanylcitratenovaplus, fentanylcitrate-dextrose, fentanylcitrate-ropivacainehcl-sodiumchloride, fentanylcitrate-sodiumchloride, fentanylnovaplus, fentanyltransdermalsystem, fentanyltroche, fentanyl/bupivacaine/epinephrine/sodiumchloride, fentanyl/bupivacaine/sodiumchloride, fentanyl/ropivacaine/sodiumchloride, fentora, hycet, hydrocodoneandchlorpheniraminepennkinetic, hydrocodonebitartrate-acetaminophen, hydrocodonebitartrate-acetaminophenavpak, hydrocodonebitartrate-chlorpheniraminemaleate, hydrocodonebitartrate-guaifenesin, hydrocodonebitartrate-homatropine, hydrocodonebitartrate-ibuprofen, hydrocodonebitartrate-phenylpropanolamine, hydrocodonebitartrate-potassiumguaiacolsulfonate, hydrocodonecp, hydrocodonehd, hydrocodonepa, hydrocodonepapediatric, hydrocodonepolistirex-chlorpheniraminepolistirex, hydrocodone-chlorpheniraminemaleate-pseudoeph, hydrocodone-phenylephrinehcl-chlorpheniramine, hydrocodone-phenylpropanolamine, hydromorphonehcl-bupivacainehcl-sodiumchloride, hydromorphonehcl-dextrose, hydromorphonehcl-ropivacainehcl-sodiumchloride, hydromorphonehcl-sodiumchloride, hysinglaer, infumorph, kadian, lazanda, lorcet, lorcet10/650, lorcethd, lortab, lortab10/325, lortab10/500, lortab2.5/500, lortab5/325, lortab5/500, lortab7.5/325, lortab7.5/500, lortabasa, lortabelixir, lortabliquid, meperedine, meperganfortis, meperidinehcl-promethazinehcl, meperidinehcl-sodiumchloride, meperitab, methadone, methadonedisp, methadonehclconcentrate, methadonehcl-sodiumchloride, methadose, methadosedisp, mitigo, morphabonder,

© 2023 Vivas-Valencia C et al. *JAMA Network Open.*

morphine, morphinesulfatecontrolledrelease, morphinesulfateimmediaterelease, morphinesulfatein5%dextrose, morphinesulfate-bupivacainehcl-sodiumchloride, morphinesulfate-sodiumchloride, morphine-dextrose, morphine-sodiumchloride, mscontin, norco, nucynta, nucyntaer, onsolis, opana, opanaer, oxaydo, oxecta, oxycodonehcl, oxycodonehcl/oxycodoneterephthalate/aspirin, oxycodonehcl-acetaminophen, oxycodonehcl-acetaminophenavpak, oxycodonehcl-aspirin, oxycodonehcl-ibuprofen, oxycontin, oxymorphonehcl, percocet, primlev, procet, propoxyphenecompound, propoxyphenecompound-64, propoxyphenehcl, propoxyphenehclcompound, propoxyphenehclw/acetaminophen, propoxyphenenapsylate, propoxyphenenapsylate/acetaminophen, propoxyphenew/aspirin&caffeine, rezira, roxicet, roxicodone, roxicodoneintensol, roxybond, sublimaze, subsys, tramadolhcl/acetaminophen, tramadolhcl/acetaminophenavpak, ultram, ultramer, verdrocet, vicodin, vicodines, vicodinhp, vicodintuss, vicodintussexpectorant, vicoprofen, vituz, xartemisxr, xodol, xodol5/300, xodol7.5/300, xtampzaer, zamicet, zohydroer, zutripro.

We excluded opioids prescribed as medication for opioid use disorder, formulations in bulk powder for compounding, or opioids containing products used for cough relief. Prescription claims were also excluded if the day's supply value was missing, zero, negative, or extremely large (i.e., 999 days).[1] We further restricted our analysis based on race/ethnicity, age, and complete demographic information (i.e., sex, and three-digit ZIP code). For race/ethnicity, we selected patients with self-report race/ethnic categories as Black, Hispanic, and White. Patients in other race/ethnic categories were excluded due to the small sample size (<2% of the analytical cohort). For age, only patients ≥18 years were included in the study (see **eFigure**). Because we did not have access to enrollment data, to ensure that patients had been continually enrolled in our dataset, we required at least one diagnosis in their claim history or any prescription activity within one year after opioid treatment discontinuation. Therefore, our sample cohort only included opioid treatments ending before September 2020. We applied this inclusion criterion so that treatment discontinuation is not misclassified because the patient's identifier no longer appears in the database due to relocation to a different state, change of insurance, or death.

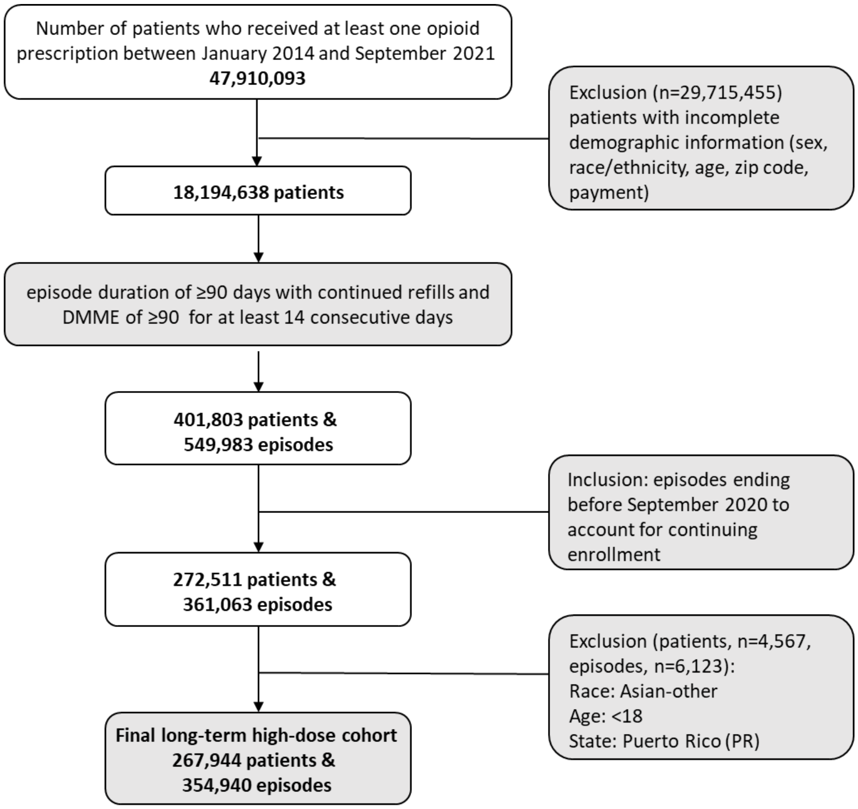

eFigure. Identification of patients and their respective episodes

© 2023 Vivas-Valencia C et al. *JAMA Network Open.*

Measurements

Opioid treatment episode

The continuity of an opioid treatment episode was indicated by the consecutive refill of prescriptions involving opioid analgesics. We defined the start of a new opioid treatment episode as the date when a prescription was filled, and we considered the episode to be discontinued if there was a gap of more than 30 days between the end of the days' supply of one prescription and the fill of a subsequent prescription.[2,3] For each prescription, we calculated the corresponding DMME by multiplying the quantity dispensed, strength per unit, and an MME conversion factor from the CDC.[4] We identified patients who received LTHD treatment, defined as episodes with ≥90 days and DMME of ≥90 for at least 14 consecutive days during an episode.[5] A period of 90 days is used consistent with current definitions for chronic conditions,[6] and DMME of 90 or more follows clinical recommendations thresholds.[7,8]

Abrupt and gradual discontinuation

The primary outcome was opioid treatment discontinuation type, i.e., abrupt vs. gradual discontinuation. We defined an episode to be gradually discontinued if the average DMME for the final 30 days of treatment was 10-34% lower than the previous 30 days.[9,10] An episode was also identified as gradually discontinued if the last 30 days of an episode and the DMME of the last two-month rolling average were <60 DMME. An episode was classified as abruptly discontinued under three instances: 1) if the DMME for the final 30 days was ≥60 with no sign of gradual discontinuation,[2] 2) if the last 30-day of the episode was <60 DMME but the two-month rolling average before discontinuation was ≥60 DMME, or 3) if the percentage reduction between the final 30 days of treatment was 35% or higher than the previous 30 days (see **eTable**). Following previous literature,[10] we applied an algorithm based on the rolling average to smooth the fluctuations in dosing attributed to gaps between refills and overlapping prescriptions.

eTable. Algorithm used to classify episodes

**Last DMME (A)**	**Prior to last DMME (B)**	**Change % A vs B**	**Type of discontinuation**
60+	60+	0	Abrupt
60+	60+	Reduce <10	Abrupt
60+	60+	Reduce 10-34	Gradual
60+	60+	Reduce 35+	Abrupt
60+	60+	Increase	Abrupt
60+	<60	Increase	Abrupt
<60	60+	Reduce <10	Abrupt
<60	60+	Reduce 10-34	Gradual
<60	60+	Reduce 35+	Abrupt
<60	<60	0	Gradual
<60	<60	Reduce <10	Gradual
<60	<60	Reduce 10-34	Gradual
<60	<60	Reduce 35+	Gradual
<60	<60	Increase	Gradual
**Last DMME (A)**	**Two-month rolling average (C)**	**Change % A vs C**	**Type of discontinuation**
<60	60+	Reduce <10	Abrupt
<60	60+	Reduce 35+	Abrupt

Explanatory variables

Patient demographic information included race (Black, Hispanic, White), sex (male, female), age (18-34, 35-49, 50-64, 65+), and the state where the episode took place. The primary payment source of an opioid treatment episode was coded as the payment source of the last prescription dispensed during an episode (cash, private insurance, Medicare, or Medicaid). Additionally, we considered the initial opioid dosage level (<50 DMME, 50-90 DMME, 90-120 DMME, >120 DMME) and the calendar year when an episode ended.

© 2023 Vivas-Valencia C et al. *JAMA Network Open.*

Analysis
Our analyses were conducted at the treatment episode level, and all the episodes were discontinued before September 2020. Therefore, episodes that started in recent years were disproportionally excluded from this study. We first estimated the number and percentage of episodes abruptly discontinued by population subgroup. Second, using national-level data, we built bivariate and multivariate logistic regressions to examine the probability of being abruptly discontinued from LTHD opioid treatment as a function of patient demographics, payment type, calendar year, and the level of DMME at the beginning of the episode. To further examine the heterogeneity of the associated factors in each state, we repeated the regression analyses using state-level data. We report adjusted odds ratios and 95% confidence intervals. All P-values were two-sided, and we used 0.05 as the significant level. Benjamini–Hochberg correction for multiple comparisons was applied. Data were analyzed using R version 4.2.0.

eReferences

1. Woods C, Chai G, Meyer T, Staffa J, Dal Pan G. Patterns of opioid analgesic use in the U.S., 2009 to 2018. *Pain*. 2021;162(4). doi:10.1097/j.pain.0000000000002101
2. Bao Y, Zhang H, Wen K, et al. Robust Prescription Monitoring Programs and Abrupt Discontinuation of Long-term Opioid Use. *Am J Prev Med*. 2021;61(4). doi:10.1016/j.amepre.2021.04.019
3. Fenton JJ, Agnoli AL, Xing G, et al. Trends and Rapidity of Dose Tapering Among Patients Prescribed Long-term Opioid Therapy, 2008-2017. *JAMA Netw Open*. 2019;2(11). doi:10.1001/jamanetworkopen.2019.16271
4. Centers for Disease Control and Prevention. Opioid National Drug Code and Oral MME Conversion File Update. Published February 1, 2023. Accessed August 28, 2023. https://www.cdc.gov/opioids/data-resources/index.html
5. Stein BD, Sherry TB, O'Neill B, Taylor EA, Sorbero M. Rapid Discontinuation of Chronic, High-Dose Opioid Treatment for Pain: Prevalence and Associated Factors. *J Gen Intern Med*. 2022;37(7). doi:10.1007/s11606-021-07119-3
6. Bernell S, Howard SW. Use Your Words Carefully: What Is a Chronic Disease? *Front Public Health*. 2016;4. doi:10.3389/fpubh.2016.00159
7. Dowell D, Haegerich TM, Chou R. CDC guideline for prescribing opioids for chronic pain-United States, 2016. *JAMA - Journal of the American Medical Association*. 2016;315(15). doi:10.1001/jama.2016.1464
8. Dowell D, Ragan KR, Jones CM, Baldwin GT, Chou R. CDC Clinical Practice Guideline for Prescribing Opioids for Pain - United States, 2022. *MMWR Recomm Rep*. 2022;71(3). doi:10.15585/mmwr.rr7103a1
9. Bao Y, Wen K, Johnson P, Witkin LR, Reid MC. Abrupt Discontinuation of Long-Term Opioid Therapies Among Privately Insured or Medicare Advantage Adults, 2011-2017. *Pain Medicine (United States)*. 2021;22(7). doi:10.1093/pm/pnaa350
10. Diprete BL, Ranapurwala SI, Maierhofer CN, et al. Association of Opioid Dose Reduction with Opioid Overdose and Opioid Use Disorder among Patients Receiving High-Dose, Long-term Opioid Therapy in North Carolina. *JAMA Netw Open*. 2022;5(4). doi:10.1001/jamanetworkopen.2022.9191

© 2023 Vivas-Valencia C et al. *JAMA Network Open.*
