# Supplementary material for: Factors Associated With Abrupt Discontinuation of Long-Term High-Dose Opioid Treatment
Source: JAMA Netw Open. 2023 Nov 3;6(11):e2341416. doi: 10.1001/jamanetworkopen.2023.41416 (PMC10625034; doi:10.1001/jamanetworkopen.2023.41416)
Supplement: Supplement 2. — Data Sharing Statement [file jamanetwopen-e2341416-s002.pdf]

Data Sharing Statement

Vivas-Valencia. Factors Associated With Abrupt Discontinuation of Long-Term High-Dose Opioid Treatment. *JAMA Netw Open*. Published November 06, 2023. doi:10.1001/jamanetworkopen.2023.41416

Data
Data available: No

Additional Information
Explanation for why data not available: We used proprietary data obtained from IQVIA and don't have permission to share patient-level data; however, we have shared data summary in the supplementary document.
